# Beyond the Rash: Exploring the Symptoms and Diagnosis of Dermatomyositis

**DOI:** 10.7759/cureus.50011

**Published:** 2023-12-05

**Authors:** Daxkumar Patel, Kush Patel, Shani Parekh, Trisha Ajwani, Savan Patel

**Affiliations:** 1 Internal Medicine, Government Medical College Baroda, Vadodara, IND; 2 Medicine, Government Medical College Baroda, Vadodara, IND; 3 Internal Medicine, Government Mecical College Baroda, Vadodara, IND

**Keywords:** dermatology, rheumatology & autoimmune diseases, inflammatory myopathy, dermatomyositis, autoimmune disorder

## Abstract

Dermatomyositis represents a rare autoimmune disorder characterized by the concurrent presentation of inflammatory myopathy and distinctive cutaneous manifestations. Herein, we present a comprehensive case report involving a 62-year-old male patient exhibiting a complex array of symptoms encompassing progressive muscle weakness, characteristic dermatological findings, and systemic involvement. This case report serves to illuminate the diagnostic intricacies inherent to dermatomyositis and underscore the imperative for a multidisciplinary approach to its effective management.

The clinical presentation of the patient featured hallmark signs such as the classic heliotrope rash, Gottron's papules, and proximal muscle weakness, all indicative of dermatomyositis. Laboratory investigations revealed elevated muscle enzyme levels and the presence of positive autoantibodies, thereby reinforcing the diagnostic framework. Imaging modalities substantiated muscular involvement, while electromyography provided definitive evidence of myopathic alterations. Notably, a muscle biopsy further corroborated the diagnostic findings. In response to these diagnostic cues, the patient was expeditiously initiated on a therapeutic regimen encompassing corticosteroids, immunosuppressants, calcium channel blockers, and a tailored physical therapy program.

This case underscores the pivotal significance of timely recognition and intervention for the treatment of dermatomyositis, thus mitigating the risk of long-term complications and enhancing the patient's overall quality of life. Moreover, it highlights the indispensability of interdisciplinary collaboration, uniting the expertise of dermatologists, rheumatologists, and neurologists, in navigating the intricacies of this intricate autoimmune disorder. We emphasize the pressing need for a comprehensive evaluation and an individualized therapeutic approach, thereby amplifying the prospects for superior patient outcomes and an improved quality of life.

## Introduction

Dermatomyositis is an uncommon autoimmune disorder characterized by inflammatory myopathy and distinctive skin findings [1]. It belongs to the spectrum of idiopathic inflammatory myopathies and is associated with systemic manifestations [2]. Dermatomyositis presents unique diagnostic challenges due to its diverse clinical features and potential overlap with other connective tissue diseases [3]. Early recognition and accurate diagnosis are pivotal for initiating appropriate treatment and preventing complications [4]. This case report elucidates the complexities involved in diagnosing and managing dermatomyositis, shedding light on the pathogenesis, clinical presentation, and therapeutic options for this intriguing condition.

## Case presentation

A 62-year-old male patient presented at our medical facility with the chief complaint of anasarca, bilateral pedal edema, as well as difficulties in walking and swallowing that had persisted for the past three months. Upon conducting a thorough examination and gathering the patient's medical history, a significant observation was the presence of skin lesions on the face, scalp, trunk, neck, and nape of the neck. These skin lesions first appeared three months ago. The patient denied any history of fever, shortness of breath, reduced urine output, or unexplained weight loss. Notably, the patient exhibited noticeable facial swelling. The dermatological manifestations observed in the patient were indicative of pruritic (itchy) and photosensitive rashes. Furthermore, there was a documented reduction in muscle strength and tone. During the evaluation of muscle function, significant pain was elicited upon movement of the elbow joint.

Diagnostic approach

**Figure 1:**
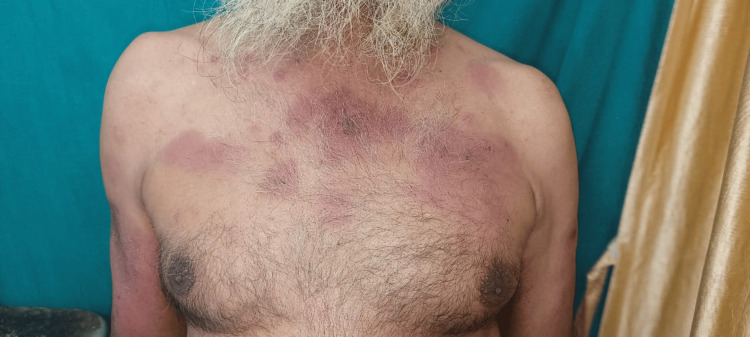
A rash over the chest indicating a shawl sign

**Figure 2:**
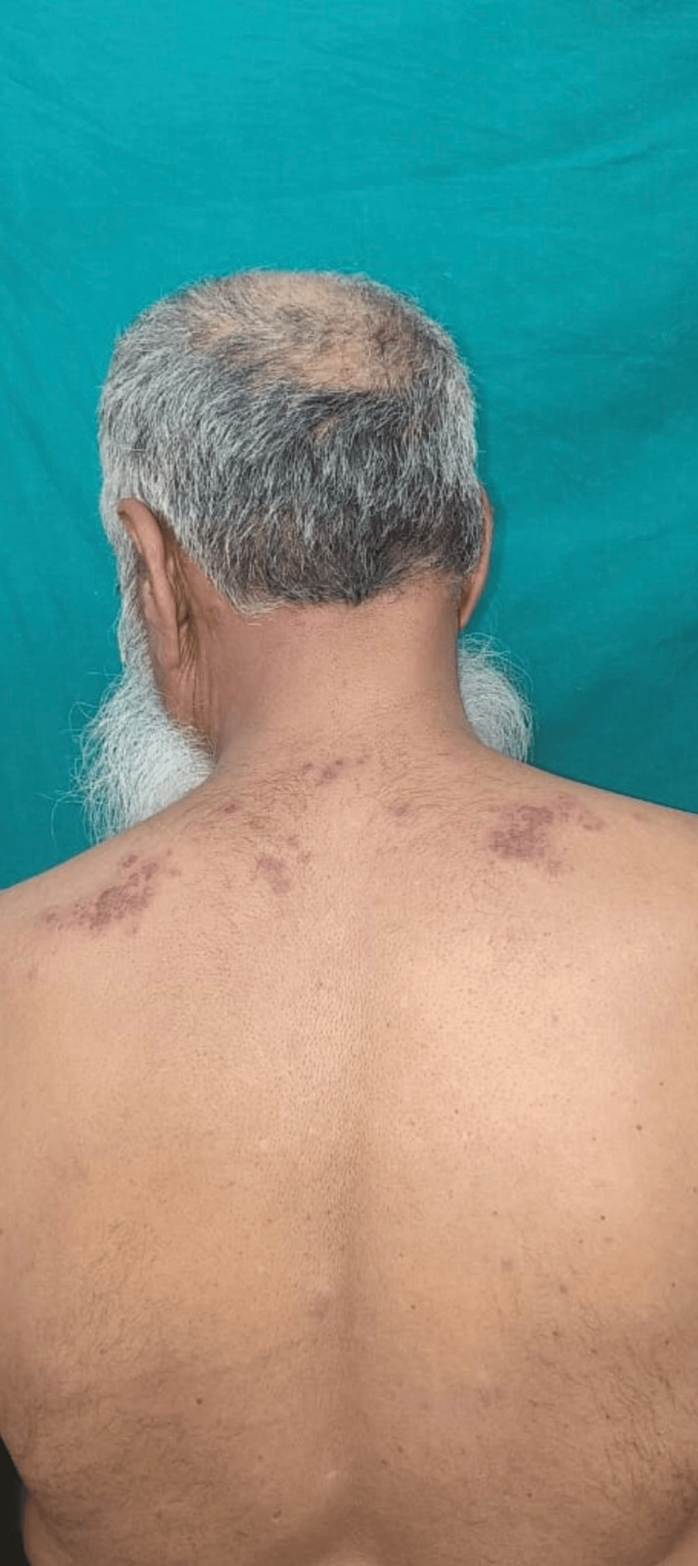
A rash on the back indicating a positive shawl sign

The physical examination revealed evident signs of inflammation, an observation further substantiated by the laboratory results, which showed an increase in both the erythrocyte sedimentation rate (ESR) and C-reactive protein (CRP). A positive antinuclear antibody (ANA) test suggested a potential autoimmune origin for the disease. Notably, the characteristic cutaneous manifestations associated with dermatomyositis were observed. This included the heliotrope rash, characterized by a violaceous or erythematous rash affecting the upper eyelids along with periorbital edema. The distinct pattern of skin involvement, marked by blackish discoloration on the face, scalp, neck, nape of the neck, and trunk, was indicative of a positive shawl sign (Figures 1-2).

Other significant cutaneous symptoms encompassed photosensitivity and pruritus.

The presence of substantial proximal muscle weakness, coupled with the heliotrope rash and positive shawl sign, strongly suggested an underlying diagnosis of dermatomyositis. Laboratory assessments indicated elevated levels of muscle enzymes, notably creatine kinase (CK) (CK total: 601.00 U/L) (CK- muscle (MM): 215.00 U/L). A muscle biopsy further revealed moderate perivascular lymphocytic infiltrates around certain perimysial and epimysial vessels, as well as perifascicular atrophy (Table 1).

**Table 1:** Radiological investigations and muscle biopsy

Investigations	Reports
Ultrasonography of the abdomen and pelvis	Normal
Muscle biopsy in formalin	The muscle biopsy shows preserved fascicular architecture with significant variation in fiber size. Myofibers are polygonal to round in shape. There are angulated fibers and atrophic fibers. Perifascicular atrophy of myofibers is noted. There are moderately perivascular lymphocytic infiltrates noted around some of the perimysial and epimysial vessels. A few myonuclear clumps are seen, and several regenerating fibers are present. Myonecrosis and myophagocytosis are evident. Signs of inflammatory myopathy are seen.

**Figure 3:**
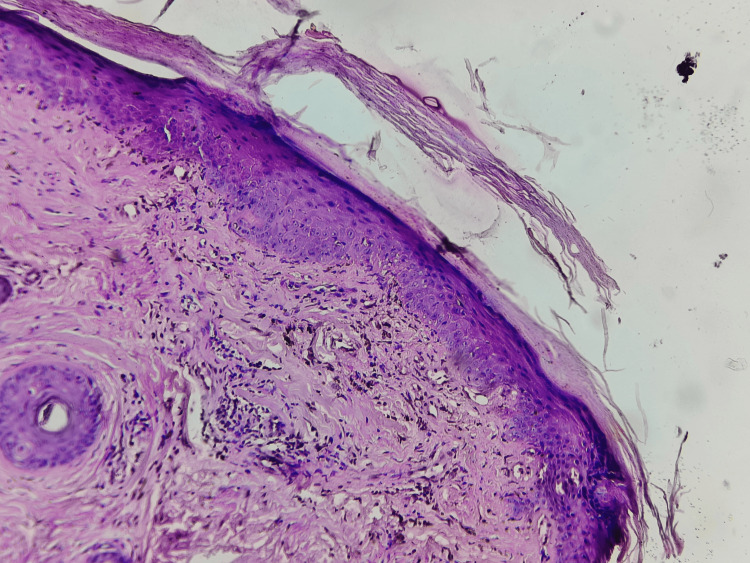
Hematoxylin and eosin (H&E)-stained slide in 10x view showing epidermal atrophy and loss of rete ridges

Hematoxylin and eosin (H&E)-stained slides showed signs of epidermal atrophy along with loss of rete ridges and dermal infiltration (Figure 3).

These findings were consistent with dermatomyositis.

Significantly, the gamma-glutamyl transferase (GGT) levels were markedly elevated (GGT = 2448.0 U/L), potentially indicating autoimmune hepatitis in light of the autoimmune nature of the condition. The negative rheumatoid factor and negative anti-cyclic citrullinated peptide (CCP) antibodies excluded the diagnosis of rheumatoid arthritis. Thyroid-stimulating hormone (TSH) levels were measured and found to be 0.99 μIU/ml (normal: 0.39-5.0 μIU/ml), ruling out any thyroid abnormalities.

In accordance with the diagnostic criteria established by Bohan and Peter in 1975 [1], which take into account the skin rashes, progressive muscle weakness, abnormal muscle biopsy results, and laboratory findings, the overall clinical picture aligned with an autoimmune condition (Table 2).

**Table 2:** Routine blood investigations with constricted-fundus examination N/L/E/M: neutrophil/lymphocyte/eosinophil/monocyte; ANA: anti-neutrophilic antibody; anti-CCP: anti-cyclic citrullinated peptide

Investigations	Values	Reference Range
Hemoglobin (gm %)	10.6	13-17 gm%
Total Count (per cumm)	7,300	4000-11000
Differential Count (N/L/E/M %)	82/09/04/05	(60%-70%)/(20%-35%)/(1%-4%)/(2%-6%)
Platelet Count (per cumm)	2,08,000	1.5-4.5 lacs/cumm
Random Blood Sugar (mg/dl)	85	70-100 mg/dl
Urea (mg/dl)	51	16.6-48.5 mg/dl
Creatinine (mg/dl)	0.9	0.6-1.2 mg/dl
Total Bilirubin (mg/dl)	4.3	0.1-1.2 mg/dl
Direct Bilirubin (mg/dl)	2.7	0.0-0.3 mg/dl
Indirect Bilirubin (mg/dl)	1.6	0.1-0.9 mg/dl
Sodium (mmol/L)	135	136-146 mmol/L
Potassium (mmol/L)	4.8	3.5-5.0 mmol/L
Magnesium (mEq/L)	1.8	1.5-2.0 mEq/L
Ionized Calcium (mmol/L)	1.31	1.16-1.31 mmol/L
Urine Routine Micro	Normal	
C-Fundus	No Papilledema	
ANA Profile	1:40 dilution, Weakly positive	
Anti-CCP Antibodies	0.8 U/mL, Negative	Negative: <5.0 , Positive: >=5.0

Thus, the conclusive diagnosis of dermatomyositis was reached.

Following the diagnosis, the patient was started on a high dose of oral prednisolone and tamsulosin, along with the administration of intravenous immunoglobulin (IVIG). With the intent to resolve a very painful complication, calcinosis, calcium channel blockers were added to the regimen. Our patient was given cilnidipine for the same. To support the treatment, vitamin D and calcium supplements were also added to the treatment regimen.

## Discussion

Dermatomyositis is classified into classical dermatomyositis, amyopathic dermatomyositis, juvenile dermatomyositis, hypomyopathic dermatomyositis, clinically amyopathic dermatomyositis, and clinically amyopathic, evolving into classic dermatomyositis [5]. While most cases of dermatomyositis involve both muscle and skin symptoms, there are variations. Clinically amyopathic dermatomyositis (CADM) is a condition where patients exhibit the typical skin manifestations of dermatomyositis but do not experience muscle weakness. Clinically amyopathic dermatomyositis can be further categorized into hypomyopathic and amyopathic dermatomyositis. Hypomyopathic dermatomyositis patients do not display clinically evident muscle weakness [6].

The patient showed typical features for the diagnosis of dermatomyositis, such as progressive muscle weakness, elevated muscle enzymes, dysphagia, and erythematous rashes all over the body with raised ESR, CRP, and a positive ANA report.

Early detection and treatment are crucial to prevent complications and minimize muscle and skin damage [1]. Treatment involves a multidisciplinary approach, including medications such as corticosteroids, immunosuppressants, calcium channel blockers, and physical therapy to manage symptoms, reduce inflammation, and improve muscle strength [7]. 

Differential diagnosis for a similar presentation of symptoms and history includes inclusion body myositis displaying asymmetric, distal weakness with atrophy, marked by inclusion bodies. Drug-induced myopathy necessitates a drug history assessment, may lack skin involvement, and demands no muscle biopsy. Hypothyroidism mirrors proximal weakness and elevated muscle enzymes, pointing towards a thorough thyroid evaluation.

## Conclusions

In conclusion, this case report highlights the clinical presentation, diagnostic journey, and management of a patient with dermatomyositis. The patient's symptoms, including proximal muscle weakness, characteristic cutaneous manifestations such as the heliotrope rash and positive shawl sign, as well as elevated muscle enzymes and autoimmune markers, collectively supported the diagnosis of dermatomyositis. The utilization of established diagnostic criteria, including those proposed by Bohan and Peter, further solidified the accuracy of the diagnosis.

The case underscores the importance of a comprehensive and multidisciplinary approach to managing dermatomyositis, involving dermatologists, rheumatologists, and other relevant specialists. Early detection and intervention are crucial to prevent potential complications and enhance the patient's quality of life. The successful management of this case involved a combination of immunosuppressive therapy, targeted skin care, physical therapy, and ongoing monitoring of disease activity and associated organ involvement.

This report not only contributes to the medical literature on dermatomyositis but also emphasizes the significance of recognizing both the dermatological and systemic aspects of the condition. It serves as a reminder that dermatomyositis is a complex autoimmune disorder that requires integrated medical attention, paving the way for improved patient outcomes and a deeper understanding of this rare yet impactful condition.
